# Impact of reproductive aging on the vaginal microbiome and soluble immune mediators in women living with and at-risk for HIV infection

**DOI:** 10.1371/journal.pone.0216049

**Published:** 2019-04-26

**Authors:** Kerry Murphy, Marla J. Keller, Kathryn Anastos, Shada Sinclair, J. Cooper Devlin, Qiuhu Shi, Donald R. Hoover, Brian Starkman, Jamie McGillick, Caroline Mullis, Howard Minkoff, Maria Gloria Dominguez-Bello, Betsy C. Herold

**Affiliations:** 1 Department of Medicine, Albert Einstein College of Medicine, Bronx, New York, United States of America; 2 Department of Epidemiology & Population Health, Albert Einstein College of Medicine, Bronx, New York, United States of America; 3 Department of Pediatrics, Albert Einstein College of Medicine, Bronx, New York, United States of America; 4 Department of Medicine, New York University School of Medicine, New York, New York, United States of America; 5 School of Health Sciences and Practice, New York Medical College, Valhalla, New York, United States of America; 6 Rutgers University, Piscataway, New Jersey, United States of America; 7 State University of New York/Downstate Medical Center School of Medicine, Brooklyn, New York, United States of America; 8 Cincinnati Children’s Medical Center, Cincinnati, Ohio, United States of America; 9 Icahn School of Medicine at Mount Sinai, New York, New York, United States of America; 10 Department of Obstetrics and Gynecology, Maimonides Medical Center, and State University of New York/Downstate Medical Center, Brooklyn, New York, United States of America; 11 Department of Biochemistry and Microbiology, and Department of Anthropology, Rutgers, The State University of New Jersey, New Brunswick, New Jersey, United States of America; 12 Department of Microbiology and Immunology, Albert Einstein College of Medicine, Bronx, New York, United States of America; Rush University, UNITED STATES

## Abstract

**Background:**

Reproductive aging may impact the vaginal microbiome and genital tract mucosal immune environment and contribute to genital tract health in women living with and at-risk for HIV infection.

**Methods:**

A cross-sectional study of 102 HIV+ (51 premenopausal, 51 postmenopausal) and 39 HIV-uninfected (HIV-) (20 premenopausal, 19 postmenopausal) women was performed in Bronx and Brooklyn, NY. Cervicovaginal lavage (CVL) was collected for quantification of innate antimicrobial activity against *E*. *coli*, HSV-2 and HIV and immune mediators by Luminex and ELISA. Microbiome studies by qPCR and 16S rRNA sequencing were performed on vaginal swabs.

**Results:**

HIV+ postmenopausal compared to premenopausal participants had lower median *E*. *coli* bactericidal activity (41% vs. 62%, p = 0.001), lower median gene copies of *Lactobacillus crispatus* (p = 0.005) and *Lactobacillus iners* (p = 0.019), lower proportions of *Lactobacillus iners*, higher proportions of *Gardnerella* and *Atopobium vaginae* and lower levels of human beta defensins (HBD-2, HBD-3) and secretory leukocyte protease inhibitor (SLPI), p<0.001. HSV-2 inhibitory activity was higher in HIV+ postmenopausal compared to premenopausal participants (37% vs. 17%, p = 0.001) and correlated with the proinflammatory molecules interleukin (IL) 6, IL-8, human neutrophil peptide (HNP) 1–3, lactoferrin and fibronectin. Similar trends were observed in HIV- postmenopausal compared to premenopausal participants. HIV inhibitory activity did not differ by reproductive status in the HIV+ participants but was significantly higher in HIV- postmenopausal compared to premenopausal participants and in participants with suppressed plasma viral load, and inversely correlated with gene copies of *G*. *vaginalis* and BVAB2. A significant proportion of HIV+ participants on ART exhibited HIV *enhancing* activity.

**Conclusions:**

HIV+ postmenopausal compared to premenopausal participants have less CVL *E*. *coli* bactericidal activity, reflecting a reduction in Lactobacilli and a greater proportion of *Gardnerella* and *A*. *vaginae*, and more HSV-2 inhibitory activity, reflecting increased mucosal inflammation. The effect of menopause on mucosal immunity was greater in HIV+ participants, suggesting a synergistic impact. Promotion of a lactobacillus dominant vaginal microbiome and reduced mucosal inflammation may improve vaginal health and reduce risk for shedding of HIV and potential for HIV transmission in HIV+ menopausal women.

## Introduction

**Fig 1:**
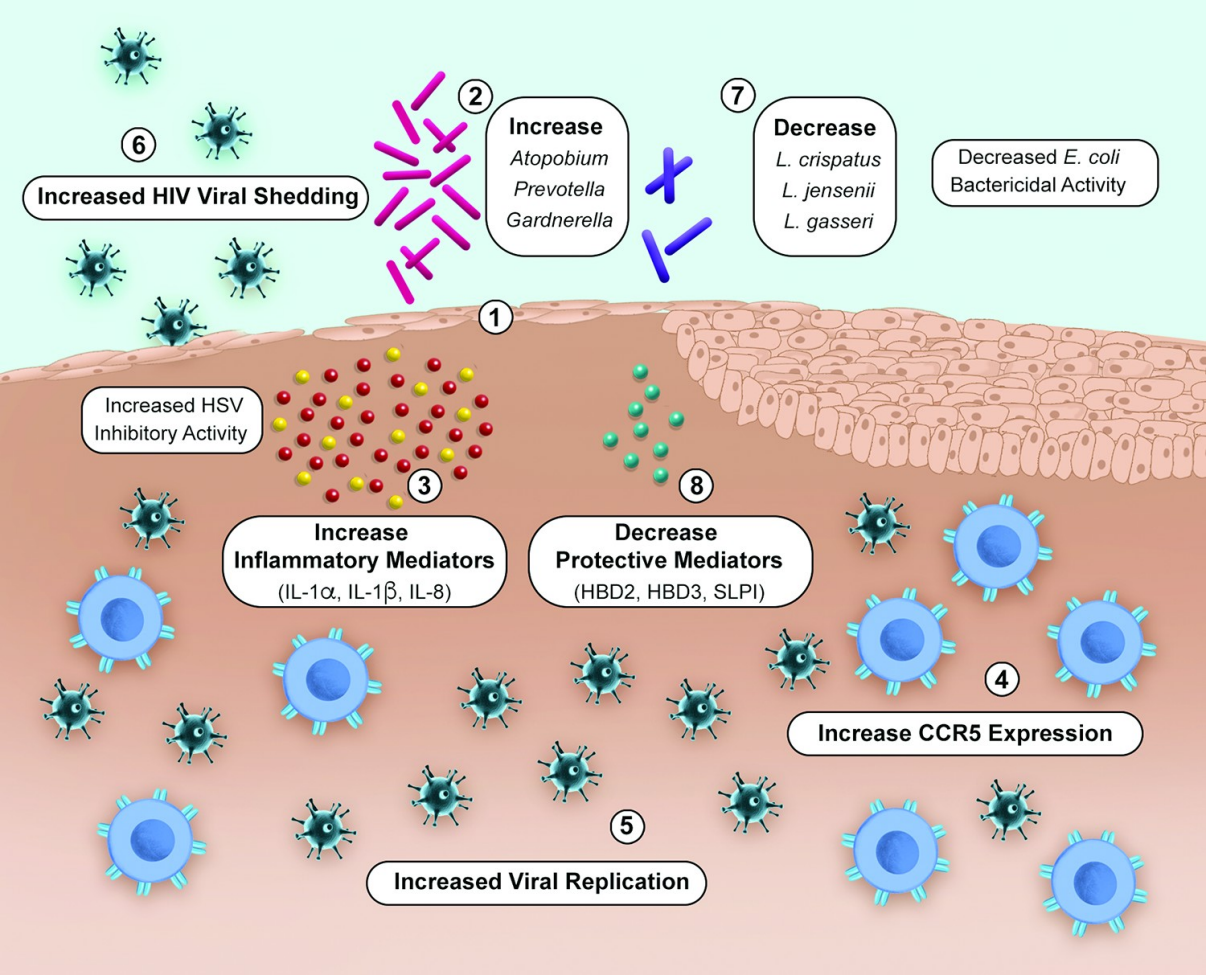
Mechanisms mediating risk for HIV acquisition and transmission in menopausal women. Numbers and corresponding labels indicate potential mechanisms. During menopause there is a loss of epithelial barrier integrity (1) and increase in BV associated species including *Atopobium*, *Prevotella*, and *Gardnerella* (2), which influence release of proinflammatory cytokines e.g. IL-1α, IL-1β and IL-8 (3) promoting recruitment and/or activation of HIV target cells (4) which may increase risk for HIV acquisition and for HIV positive women increase HIV replication (5) and subsequent viral shedding (6). Loss of H_2_0_2_ producing protective lactobacillus species *Lactobacillus (L*.*) crispatus*, *L jensenii*, *L*. *gasseri* (7) and decreased protective immune mediators (human beta defensins, SLPI) (8) may also increase risk for HIV acquisition during menopause. In menopausal women with HIV, *E*. *coli* antibacterial activity is lower, reflecting a *Lactobacillus* deficient microbiome and HSV inhibitory activity is higher reflective of inflammation.

Menopause may be a time of reduced genital tract health reflecting changes in the vaginal microbiome and mucosal environment. Previous studies in HIV uninfected (HIV-) women have demonstrated a reduction in lactobacilli with an increase in diverse anaerobes in postmenopausal compared to premenopausal women.[1, 2] Changes in immune cell phenotype and function, disruption of epithelial integrity, reduction in protective immune mediators and increased expression of pro-inflammatory genes have also been described.[1–5] Together, these changes may increase the risk of acquiring HIV and, in HIV-infected (HIV+) women, promote viral shedding and transmission (Fig 1).[6–10] Among incident US HIV infections in 2015, 17% occurred in persons >50 years of age and approximately 45% of people living with HIV in the US are aged ≥50, [11, 12] underscoring the importance of studying the impact of menopause on the cervicovaginal mucosal environment and its link to HIV acquisition and transmission.

Genital tract secretions exhibit variable antimicrobial activity when mixed *ex vivo* with bacteria or viruses and this antimicrobial activity may serve as functional biomarkers of genital tract immunity. In healthy HIV- women, higher *E*. *coli* bactericidal activity was associated with a lactobacillus dominant microbiome and inversely with *E*. *coli* colonization.[13–15] In contrast, genital tract herpes simplex virus type 2 (HSV-2) inhibitory activity correlated with concentrations of proinflammatory cytokines, lactoferrin, lysozyme and human neutrophil peptides (HNP) 1–3, suggesting that HSV inhibitory activity may be a marker of inflammation.[16–18] Precisely what mediates genital tract HIV inhibitory activity has not been well defined and both HIV inhibitory and enhancing activity have been observed.

The genital tract mucosal environment in HIV+ women, particularly in the setting of menopause, has not been well studied. Compared to HIV- women, HIV+ women with plasma viral loads (plasma VL) >10,000 copies/ml had higher levels of mucosal proinflammatory cytokines, higher Nugent scores, and less *E*. *coli* bactericidal activity. However, no differences were observed between HIV- women and HIV+ women with low plasma VL, no comparisons were made by reproductive status and microbiome studies were not performed.[19]

Building on this foundation, we hypothesized that compared to premenopausal women, postmenopausal women would have lower *E*. *coli* bactericidal activity reflecting loss of protective lactobacilli and increased bacterial diversity and higher HSV inhibitory activity reflecting inflammation. Given the dynamic interplay between the vaginal microbiome and inflammatory molecules,[6, 20] we further hypothesized that changes in the mucosal environment and vaginal microbiome would be more pronounced in HIV+ postmenopausal women, who may have chronic immune activation and increased inflammation which may contribute to ongoing low level viral replication [21–24] and potential for genital tract shedding of HIV (Fig 1). To test this hypothesis, we conducted a cross-sectional study to compare levels of soluble mucosal immune mediators and the vaginal microbiome in HIV+ and HIV- pre and postmenopausal women.

## Materials and methods

### Study design

This study was approved by the Einstein Institutional Review Board, Approval #13-08-149. Written and oral informed consent were obtained from all participants.102 HIV+ (51 premenopausal, 51 postmenopausal) and 39 HIV- (20 premenopausal, 19 postmenopausal) participants were recruited from the Bronx and Brooklyn Women’s Interagency HIV Study (WIHS) (n = 107) or clinics associated with Montefiore and Jacobi Medical Centers (n = 34). Eligible participants were approached during the course of their semi-annual core WIHS visits, regular clinic visits or by phone. The WIHS, a prospective observational study of HIV+ and at-risk HIV- women has been previously described.[25, 26] Participants were defined as premenopausal or postmenopausal based on menstrual cycle history and confirmed with serum estradiol and follicle stimulating hormone (FSH) levels.[27–29] Exclusion criteria included pregnancy or breastfeeding, use of hormonal contraceptives, hormone replacement or medications known to suppress ovulation or menstruation in the prior 6 months, cancer, hysterectomy or bilateral salpingoophorectomy, autoimmune or inflammatory bowel disease. Clinical and demographic data including self-reported race/ethnicity and self-reported adherence to antiretroviral therapy (ART) were obtained from the most recent WIHS questionnaire and from similar questionnaires for non-WIHS participants. Estradiol, FSH, and progesterone were measured using chemoluminescent immunoassays at Montefiore Medical Center. Vaginal swabs were obtained for measurement of vaginal pH, Nugent scores, and microbiome studies followed by collection of cervicovaginal lavage (CVL) in 10 ml sterile water for quantification of antimicrobial activity and soluble immune mediators. CVL was collected in water because human beta defensin activity is salt sensitive[30, 31]; pilot studies in our lab demonstrated that the *in vitro* antimicrobial activity is similar whether CVL is collected in saline, phosphate buffered saline or water. All samples were collected according to WIHS protocols and all laboratory studies were conducted in the same laboratory and performed uniformly on specimens regardless of recruitment site.

### Laboratory methods

Total CVL protein concentration was measured by Micro BCA Protein Assay Kit (Thermo Scientific). The concentration of interleukin (IL)-1α, IL-1β, IL-6, IL-8, IL-12-p70, IL-17, interferon gamma-induced protein 10 (IP-10), macrophage inhibitory protein (MIP)-1α, MIP-1β, and tumor necrosis factor (TNF) were determined by Luminex with beads from Chemicon International and analyzed using StarStation (Applied Cytometry Systems). Secretory leukocyte protease inhibitor (SLPI) (R&D Systems), human neutrophil peptides 1–3 (HNP1-3) (Hycult Biotech) and human beta defensin-2 and 3 (HBD-2, HBD-3) (Alpha Diagnostics), lactoferrin (LTF), syndecan 3 (SDC3), fibronectin, S100A9 (calgranulin B), serine protease inhibitor Kazal-type 5 (SPINK5), and the complement protein, C5α, were determined in CVL by enzyme-linked immunosorbent assay (ELISA). We chose mediators with importance for HIV risk based on anti-viral (HBD-2 HBD-3, MIP-1α, MIP-1β, TNF) and pro-inflammatory properties (IL-1α, IL-1β, IL-6, IL-8), as well as proteins important for mucosal structural integrity (SPINK5, fibronectin) which is disrupted with reproductive aging. S100A9, fibronectin and C5α were chosen as they were elevated in participants with HIV-enhancing CVL in pilot studies in our lab. Syndecan 3 was chosen for its role in tethering HIV target cells. Luminex assays were performed with undiluted samples and ELISAs assays with diluted samples based on prior studies.[19] Concentrations below the lower limit of detection (LLOD) were set at the midpoint between zero and the LLOD and corrected for dilution as indicated. To assess the bactericidal activity of CVL against *E*. *coli*, bacteria (ATCC strain 4382627 or clinical isolates) were grown overnight to stationary phase and then approximately 10^9^ cfu/ml were mixed with CVL (diluted 2X with normal saline) or control buffer and incubated at 37°C for two hours. The mixtures were then further diluted 1000X to yield 800–1000 colonies and plated in duplicate on agar enriched with trypticase soy broth and colony forming units (cfu) counted after an overnight incubation at 37°C. To measure endogenous HSV inhibitory activity, Vero cells were infected with 50–200 plaque forming units (PFU) of HSV-2(G) mixed 1:1 with CVL or control buffer and plaques counted after 48 hours. To measure endogenous HIV inhibitory activity, TZM-bl cells were infected with HIV-1_Bal_ (approximately 10^3^ TCID_50_) mixed 1:1 with CVL or control buffer and after 48 hr incubation, viral infection measured using a luciferase assay. Data are presented as median percent reduction of *E*. *coli* colony forming units (CFU), HSV plaques, or HIV luciferase activity relative to control buffer.[17, 19]

CVL HIV RNA levels were determined by centrifuging CVL at 700g and quantifying HIV RNA in the supernatants using the Abbott m2000 HIV-1 RealTime System with a LLOD of 40 copies. Plasma VL and CD4 count were obtained from WIHS database (n = 76), most recent plasma VL or CD4 count available from primary care physician (n = 22) or using the Abbott RealTime HIV-1 assay (n = 4).

### Evaluation & analysis of the microbiome

Total DNA was extracted using MoBio PowerSoil kit, and the V4 region of the 16S rRNA gene was amplified using the Illumina-adapted universal primers 515F/806R. Amplicons were combined in equimolar ratios, purified and sequenced on the Illumina MiSeq platform. Analysis of sequencing data was performed using QIIME v1.9.1.[32] Paired end sequences were joined and demultiplexed before open reference OTU picking with default parameters and using Greengenes database v13.8.[33] The OTU table generated was then filtered to remove samples with less than 500 OTUs. The OTU table summary and the full script used to process our data are available in supporting information. Sample diversity was evaluated using weighted and unweighted Unifrac metrics and visualized by PCoA to observe clustering by reproductive status, race, *E*. *coli*, HSV and HIV inhibition in HIV+ and HIV- women and by ART regimen for HIV+ participants. Significance of these observations were determined by an ANOSIM test with 999 sample permutations. Samples were rarefied at 7,694 reads and an ANOVA test with post-hoc Tukey was employed to test for significant differences in alpha diversity metrics. LefSe analysis was used to identify microbial biomarkers for the different subpopulations of samples, with LDA score threshold of 2.0. Taxa overrepresented in a subpopulation are indicated by an asterisk.

Quantitative 16S rRNA PCR (qPCR) was performed using primers and a dual-labeled fluorogenic probe hydrolyzed during PCR specific for each bacterium’s 16S rRNA gene as previously described[34] for *L*. *crispatus*, *L*. *jensenii*, *L*. *iners*, *G*. *vaginalis*, bacterial vaginosis associated bacteria BVAB2 and *Prevotella bivia* using previously published primer and probe sequences.[34, 35] Plasmid standards for *G*. *vaginalis*, BVAB2, *L*. *crispatus* and *L*. *jensenii* were gifted by David Fredricks. Genomic DNA was extracted from ATCC strains of *L*. *iners* (55195) and *Prevotella bivia* (29303) and used to perform digital PCR for determination of gene copies/volume for use as qPCR standards. LLOD for *L*. *jensenii* and *G*. *vaginalis* was 50 gene copies/swab, for *L*. *crispatus* and BVAB2 LLOD 25 gene copies/swab and for *L*. *iners* and *Prevotella bivia* 605 and 97 gene copies/swab, respectively. We chose to study *L*. *crispatus* and *L*. *jensenii* as they are known to be protective H_2_O_2_-producing lactobacilli, *L*. *iners* as it is highly prevalent in women of African descent, and *G*. *vaginalis*, BVAB2, and *P*. *bivia* as they are associated with bacterial vaginosis (BV), genital inflammation and HIV-1 viral shedding.

### Statistical analyses

Concentrations of mediators were log_10_transformed to reduce skewness in the data. Values for mediators with a significant percentage of samples below the LLOD were dichotomized at the LLOD. Categorical variables were compared between groups by Fisher exact test and continuous variables were compared by ANOVA. Spearman correlation coefficients were calculated to assess for associations between all variables and *E*. *coli*, HSV and HIV inhibitory activity. Rho (r) values of ≥0.30 were reported as these were considered to be of modest clinical significance. Two-sided p values ≤ 0.05 were considered to be statistically significant. To truncate the influence of outliers from variables, all values ≥3 standard deviations (SD) above the median were Winsorized to the median + 3 SD. If the distributions still had skewness greater than 3 after that, then variables were log transformed to stabilize the distribution. Corrections for multiple comparisons were not done due to the exploratory nature of the study and modest sample sizes. Analyses were performed in SAS, version 9.4. Figures were constructed using R 3.4.2 “Single Candle” and GraphPad Prism, version 7.

## Results

### Clinical characteristics of study participants

Postmenopausal women, independent of HIV status, were significantly older and were more likely to be HSV-2 and hepatitis C (HCV) seropositive. Smoking was more prevalent in the HIV- compared to the HIV+ women as previously reported in WIHS.[36] In both HIV+ groups, 90% of participants reported ART use and more than 80% reported ≥ 95% adherence to ART. The majority had CD4 counts >500 cells/μL (6/102 participants had CD4 counts <200 cells/μL), more than 70% had undetectable HIV-1 plasma VL, and over 95% of those studied had undetectable CVL HIV-1 RNA (Table 1).

**Table 1:** Demographic and clinical characteristics for all participants.

	HIV+ Pre (n = 51)	HIV+ Post (n = 51)	p	HIV- Pre (n = 20)	HIV- Post (n = 19)	p	p value across HIV status
**Age, years**	41.4 (36.5, 46.8)	55.9 (54.3, 58.8)	**<0.001**	44.7 (37.1, 46.4)	55.6 (54.5, 57.3)	**<0.001**	0.94
**Race**							
Black	34 (66.7%)	38 (74.5%)	0.53	11 (55%)	14 (73.7%)	0.57	0.57
White	9 (17.6%)	5 (9.8%)		3 (15%)	2 (10.5%)		
Other	8 (15.7%)	8 (15.7%)		6 (30%)	3 (15.8%)		
**Current Smoker**	21 (41.2%)	17 (33.3%)	0.54	12 (60%)	12 (63.2%)	>0.99	**0.01**
**HSV-2** **Seropositive**	40 (81.6%)	50 (100%)	**0.001**	14 (70%)	19 (100%)	**0.02**	0.36
**HCV Infection**	3 (5.9%)	23 (46%)	**<0.001**	1 (5%)	8 (42.1%)	**0.008**	0.83
**Nugent score**	5.0 (1.5, 8)	5.0 (4, 7)	0.46	6.0 (1,8)	4.0 (3,7)	0.89	0.89
**Vaginal pH**	5.2 (4.9,5.5)	5.2 (5.0,5.5)	0.08	5.2 (4.75,5.75)	5.2 (4.9,5.5)	0.77	0.95
**Estradiol (pg/ml)**	63.15 (25.2,93.4)	20 (20,20)	**<0.001**	60.2 (25.5,92.7)	20 (20, 21.7)	**<0.001**	0.98
**FSH (mIU/ml)**	4.6 (3.7,8.8)	49.8 (36,70)	**<0.001**	5.95 (4.5,10)	38.5 (29,59)	**<0.001**	0.18
**Current ART use**	46 (90.2%)	46 (90.2%)	>0.99				
**Adherence** ≥ **95%**	38/46 (82.6%)	43/46 (93.5%)	0.20				
**PVL** **Undetectable**	37 (72.6%)	36 (70.6%)	>0.99				
**CVL VL** **Undetectable**	28/29 (96.6%)	43/45 (95.6%)	>0.99				
**CD4 >500** **cells/μl**	29/49 (59.2%)	35/51 (68.6%)	0.4				

Categorical variables reported as n (%) and continuous variables reported as median (25th%,75th%). p values are reported between HIV+ pre and postmenopausal participants, HIV- pre and postmenopausal participants and between all HIV- and HIV+ participants.

### Reduction in *E*. *coli* bactericidal activity in postmenopausal HIV+ women correlates with microbiome changes

**Fig 2:**
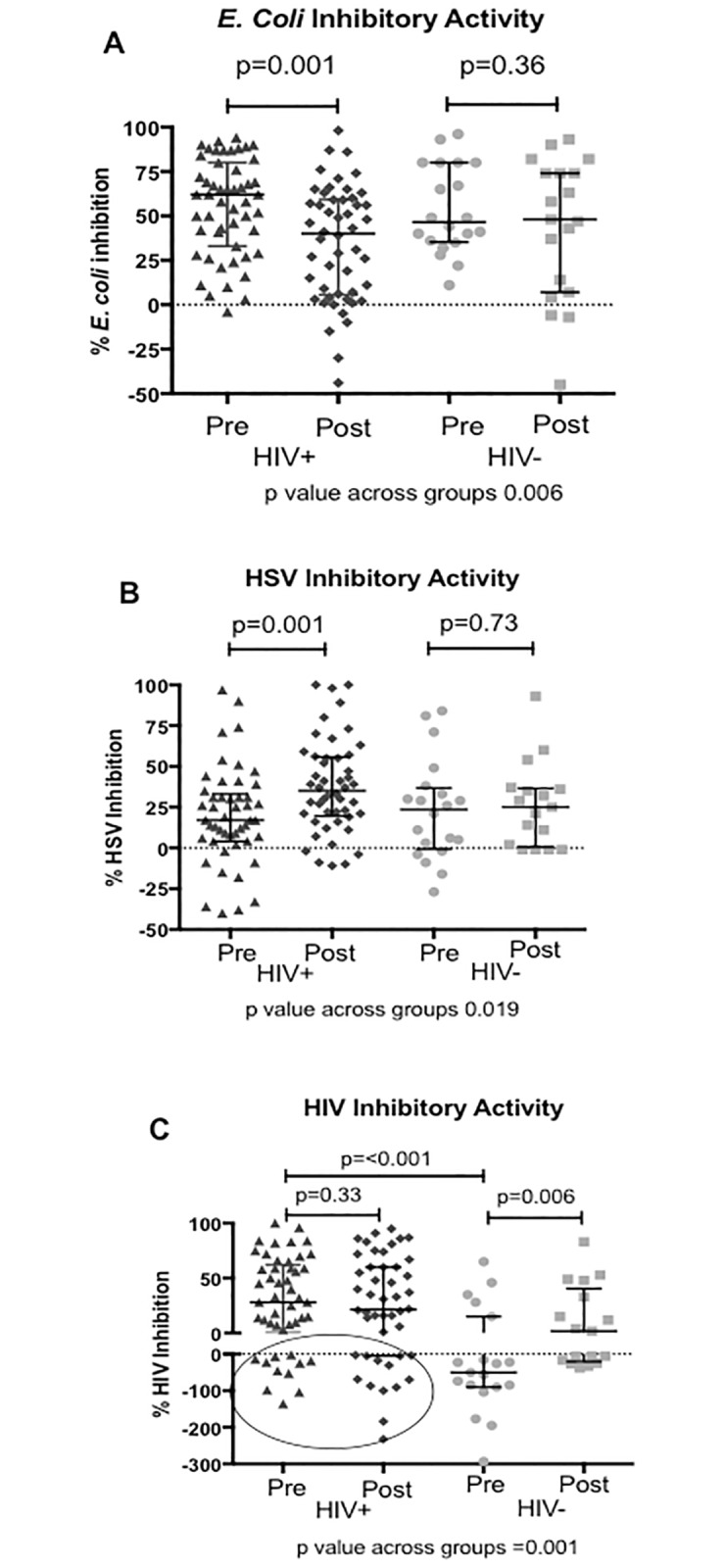
HIV+ postmenopausal participants have significantly lower CVL *E*. *coli* and higher HSV inhibitory activity. CVL *E*. *coli* (A), HSV (B) and HIV inhibitory activity (C) in premenopausal and postmenopausal HIV negative and HIV positive participants. The lines represent the median with interquartile range for percent inhibition of *E*. *coli* colonies (A) percent inhibition of HSV plaques (B) and percent reduction in relative luciferase units compared with control (C). Comparisons were made between all pairwise groups, only those with p values ≤ 0.05 and those comparing pre and postmenopausal participants are reported. Circle indicates HIV enhancing activity in HIV+ participants on ART.

**Fig 3:**
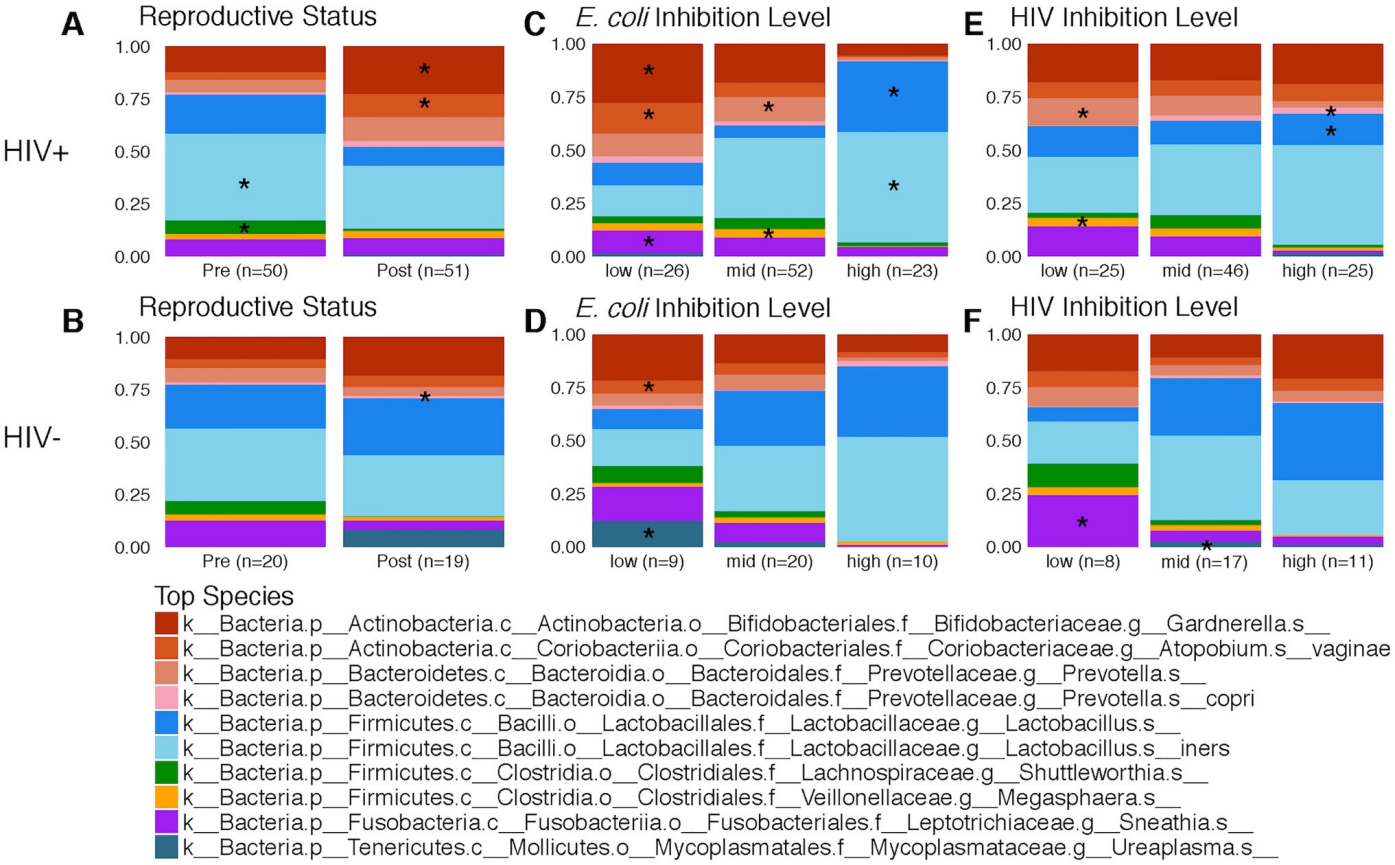
Differences in the taxonomic composition of the vaginal microbiome by reproductive status and levels of *E*. *coli* and HIV inhibitory activity. Taxonomic composition of the vaginal microbiome by reproductive status for HIV+ (A) and HIV- (B) participants, by levels of CVL *E*. *coli* bactericidal activity for HIV+ (C) and HIV- (D) participants and by levels of CVL HIV inhibitory activity for HIV+ (E) and HIV- (F) participants. Proportion of genera contributing >1% shown. Asterisks (*) indicate taxa with significantly different relative abundances between groups as determined by an LDA score ≥2. Low and high *E*. *coli* and HIV inhibition correspond to the bottom and top quartiles.

The median (IQR) % CVL *E*. *coli* bactericidal activity was significantly lower in the postmenopausal compared to premenopausal HIV+ women (41% [6,60] versus 62% [33,80] (p = 0.001), but no significant changes were observed for HIV- women (Fig 2A). *E*. *coli* bactericidal activity correlated positively with gene copies of *L*. *jensenii* (rho 0.34) and negatively with Nugent score (rho -0.3) and vaginal pH (rho -0.4) (Table 2). When analyzing the taxonomic composition of the vaginal microbiome based on levels of *E*. *coli* bactericidal activity, HIV+ participants with the highest quartile of *E*. *coli* bactericidal activity had significantly greater proportions of lactobacillus and those with the lowest quartile of *E*. *coli* bactericidal activity had significantly higher proportions of BV associated species, including *Gardnerella* and *Atopobium vaginae* (Fig 3C). There was a strong inverse correlation between *E*. *coli* bactericidal activity and alpha diversity driven by lower diversity in the participants with highest *E*. *coli* bactericidal activity (S1 Fig). *E*. *coli* bactericidal activity also positively correlated with SLPI, HBD-2, HBD-3, S-100-A9, SPINK5, and IP-10 (rho 0.3–0.47 range) (Table 2).

**Table 2:** Associations of antimicrobial activity with select immune mediators and vaginal bacteria among all participants.

	rho	p
***% E*. *coli inhibition***		
Nugent score	-0.3	<0.001
Vaginal pH	-0.4	<0.001
Log_10_ SLPI	0.42	<0.001
Log_10_ HBD-2	0.44	<0.001
Log_10_ HBD-3	0.28	<0.001
Log_10_ S100-A9	0.47	<0.001
Log_10_ SPINK5	0.38	<0.001
IP-10	0.36	<0.001
*L*. *jensenii* gene copies	0.34	<0.001
***% HSV Inhibition***		
Log_10_ HNP 1–3	0.33	<0.001
Log_10_ Lactoferrin	0.34	<0.001
Log_10_ IL-6	0.32	0.004
Log_10_ IL-8	0.44	<0.001
Log_10_ Fibronectin	0.33	<0.001
***% HIV Inhibition***		
IL-1α	-0.34	<0.001
*G vaginalis* gene copies	-0.26	0.002
BVAB-2 gene copies	-0.30	<0.001

Spearman correlation coefficients reported as rho with associated p values

### CVL HSV-2 inhibitory activity is higher and protective immune mediators lower in postmenopausal HIV+ women

The median (IQR) % HSV-2 inhibitory activity was significantly higher, 37% (21,56) in the postmenopausal compared to 17% (4,31) in the premenopausal HIV+ women (p = 0.001), but similar in pre and postmenopausal HIV- participants (Fig 2B). There were no differences in HSV inhibitory activity based on HSV-2 serostatus. Consistent with prior studies,[16–18], the CVL HSV inhibitory activity correlated positively with IL-6, IL-8, HNP 1–3, lactoferrin, fibronectin, (all rho>0.32), but no correlations with the microbiome were observed (Table 2). The log concentration of HNP 1–3 trended high and, paradoxically, IL-6 was lower in the HIV+ postmenopausal compared to premenopausal women, but there were no differences observed for other inflammatory mediators (Table 3). Postmenopausal compared to premenopausal HIV+ women also exhibited significantly lower levels of HBD2, HBD3 and SLPI, as well as the serine proteinase inhibitor, SPINK5. Similar differences or trends were observed comparing pre and postmenopausal HIV- women.

**Table 3:** Concentrations of mucosal immune mediators in HIV+ and HIV- premenopausal and postmenopausal women.

	HIV+ Pre (n = 51)	HIV+ Post (n = 51)	p	HIV- Pre (n = 20)	HIV- Post (n = 19)	p	p value across HIV Status
**Log_10_ Total protein (μg/ml)**	2.32 (0.31)	2.21 (0.29)	0.07	2.33 (0.29)	2.25 (0.27)	0.32	0.71
**Log_10_ IL-1α (pg/ml)**	1.98 (0.61)	1.78 (0.72)	0.28	2.06 (0.53)	1.80 (0.65)	0.28	0.77
**IL-1β> LLOD (pg/ml)**	51% (25)	45.1% (23)	0.69	60% (12)	52.6% (10)	0.75	0.45
**Log_10_ IL-6 (pg/ml)**	0.54 (0.60)	0.05 (0.45)	**<0.001**	0.08 (0.46)	0.08 (0.55)	0.98	0.23
**Log_10_ IL-8 (pg/ml)**	2.02 (0.73)	1.96 (0.88)	0.80	1.98 (0.84)	1.77 (0.81)	0.51	0.49
**IL-12 p70 > LLOD (pg/ml)**	20.4% (10)	23.5% (12)	0.81	20% (4)	15.8% (3)	>0.99	0.65
**IL-17 > LLOD (pg/ml)**	6.1% (3)	3.9% (2)	0.67	10% (2)	5.3% (1)	>0.99	0.69
**IP-10 > LLOD (pg/ml)**	65.3% (32)	58.8% (30)	0.54	60% (12)	63.2% (12)	>0.99	>0.99
**MIP-1α> LLOD (pg/ml)**	10.2% (5)	15.7% (8)	0.55	5% (1)	15.8% (3)	0.34	0.78
**MIP-1β> LLOD (pg/ml)**	49% (24)	52.9% (27)	0.84	45% (9)	42.1% (8)	>0.99	0.46
**TNF > LLOD (pg/ml)**	16.3% (8)	21.6% (11)	0.61	25% (5)	21% (4)	>0.99	0.64
**C5α> LLOD (pg/ml)**	40.8% (20)	45.1% (23)	0.69	47.4% (9)	47.4% (9)	>0.99	0.70
**Log_10_ HNP 1–3 (pg/ml)**	4.45 (1.18)	4.90 (1.16)	0.06	3.97 (1.46)	4.26 (1.56)	0.55	**0.02**
**Log_10_ Lactoferrin (ng/ml)**	2.86 (0.52)	2.97 (0.67)	0.36	2.77 (0.50)	2.76 (0.73)	0.97	0.19
**Log_10_ Fibronectin (ng/ml)**	0.39 (0.96)	0.38 (0.94)	0.98	0.52 (1.03)	0.43 (0.92)	0.78	0.62
**Log_10_ HBD-2 (pg/ml)**	2.51 (0.81)	1.95 (0.62)	**<0.001**	2.72 (0.87)	1.98 (0.63)	**0.004**	0.37
**Log_10_ HBD-3 (pg/ml)**	1.97 (0.50)	1.53 (0.24)	**<0.001**	2.13 (0.55)	1.49 (0.23)	**<0.001**	0.44
**Log_10_ SLPI (pg/ml)**	4.67 (1.13)	3.68 (0.92)	**<0.001**	4.53 (0.99)	3.90 (1.05)	0.06	0.82
**Log_10_ SDC3 (ng/ml)**	0.67 (0.96)	0.88 (0.82)	0.25	0.64 (0.94)	0.76 (0.66)	0.66	0.64
**Log_10_ S100-A9 (pg/ml)**	2.16 (0.53)	2.14 (0.51)	0.86	2.32 (0.60)	2.24 (0.45)	0.64	0.18
**Log_10_ SPINK5 (ng/ml)**	2.59 (0.29)	2.42 (0.27)	**0.007**	2.64 (0.36)	2.49 (0.25)	0.17	0.30

[% (n) >LLOD or log mean (S.D.) as indicated]

### HIV activity is variable and correlates with measures of HIV control and the microbiome

HIV+ women had higher CVL HIV inhibitory activity compared to HIV- women, but there were no differences within the HIV+ women based on reproductive status (Fig 2C). In contrast, HIV- postmenopausal women had significantly higher median % (IQR) HIV inhibitory activity 2% (-16,33) compared to HIV- premenopausal women -50% (-90,15) (p = 0.006). Overall there was little HIV inhibitory activity observed and CVL from a subset of both HIV+ and HIV- women enhanced HIV infection when added to virus and cells *in vitro* (Fig 2C). The HIV inhibitory activity correlated negatively with IL-1α (rho = -0.34) and with gene copies of BVAB2 (rho = -0.30) and *G*. *vaginalis* (rho = -0.26), suggesting an association between enhancement of HIV infection and vaginal dysbiosis (Table 2). This is further supported by the finding that HIV+ women with the lowest quartile of HIV inhibition (e.g. HIV enhancing CVL) had a significantly greater proportion of *Prevotella* and *Megasphera* whereas HIV+ women with the highest quartile of HIV inhibition had a greater proportion of *Lactobacillus* (Fig 3E). Median HIV inhibitory activity was significantly lower in participants with a detectable vs. undetectable plasma VL (7 vs. 38.5, p = 0.006) and in participants not taking ART vs. ART users (-5.5 vs. 32, p = 0.015).

The observation that CVL from a subset of HIV+ women reporting high adherence to ART enhanced HIV growth *ex vivo* suggested the possibility that the differences might reflect the pharmacokinetics of the ART regimens and concentrations of drug in cervicovaginal fluid.[37] In subgroup analyses of HIV+ participants, comparing the 20 participants with the highest HIV inhibition to the 20 with the lowest HIV inhibition (enhancers), those with the lowest HIV inhibitory activity were more likely to be on a protease inhibitor (PI) (50% vs. 15%, p = 0.04) and less likely to be on an non-nucleoside reverse transcriptase inhibitor (NNRTI) based ART regimen (10% vs. 60%, p = 0.002). Participants with the lowest HIV inhibitory activity were also more likely to have a detectable plasma VL (47% vs. 11%, p = 0.027) and to have a higher median Nugent score (7 vs. 4, p = 0.02) although there were no differences by ART regimen in alpha diversity of the microbiome. Beta diversity differed significantly by ART regimen however there was no clustering observed. (S2 Fig). Overall, these findings suggest a role for antiretrovirals and the vaginal microbiome in HIV inhibitory activity.

### HIV+ postmenopausal women have lower lactobacillus and higher BV associated species

Consistent with the median vaginal pH > 5 and median Nugent score > 4 across all groups, low median copies/swab of *L*. *crispatus* (433–7094) and *L*. *jensenii* (50) and high copies of *L*. *iners* (1.2 x 10^4^–5.4 x 10^6^) and *G*. *vaginalis* (1.07 x 10^6^–2.49 x 10^6^) were recovered in vaginal swabs by qPCR (Table 4). There were no differences in quantities of bacteria recovered by HIV status, but there was a significantly lower number of gene copies per swab of *L*. *crispatus* (median 433 vs. 703, p = 0.005) and *L*. *iners* (1.2 x 10^4^ vs. 5.4 x 10^6^, p = 0.019) recovered in HIV+ postmenopausal compared to premenopausal women. The taxonomic composition of the vaginal microbiome differed by reproductive status in the HIV+ participants and was driven by significantly higher proportions of *Gardnerella* and *Atopobium vaginae* in the postmenopausal and higher proportions of *L*. *iners* and *Shuttleworthia* in premenopausal participants (Fig 3A). HIV- postmenopausal compared to premenopausal participants had a significantly lower number of gene copies of *L*. *iners* (1.7 x 10^4^ vs. 2.4 x 10^6^ p = 0.046) however were also more likely to have detectable *L*. *crispatus* compared to premenopausal women (18/19 vs. 12/20, p = 0.019). As opposed to the HIV- participants, there were significant differences in alpha and beta diversity based on reproductive status in the HIV+ participants although relatively modest and the latter did not cluster based on reproductive status (S1 and S3 Figs). Further there were no differences by self-reported race in the alpha or beta diversity of the vaginal microbiome. (S4 Fig).

**Table 4:** qPCR concentrations of bacteria from vaginal swabs.

	HIV+ Pre (n = 51)	HIV+ Post (n = 51)	p	HIV- Pre (n = 20)	HIV- Post (n = 19)	p	p value across HIV Status
***L*. *crispatus***							
% (No.) PCR +	68.6% (35)	70.6% (36)	>0.99	60% (12)	94.7% (18)	**0.019**	0.53
Gene copies/swab, median (IQR)	703 25, 2.8x10^5^	433 25, 1.3x10^4^	**0.005**	692 25, 4,4x10^4^	7094 356, 8.0x10^5^	0.51	0.13
***L*. *jensenii***							
% (No.) PCR+	47% (24)	31.3% (16)	0.15	45% (9)	42.1% (8)	>0.99	0.70
Gene copies/swab	50 50, 6.3x10^5^	50 50, 335	0.08	50 50, 2.0x10^4^	50 50, 2.4x10^3^	0.3	0.32
*L*. iners*							
% (No.) PCR+	88.9% (40/45)	69.7% (30/43)	**0.035**	94.4% (17/18)	61.1% (11/18)	**0.04**	0.81
Gene copies/swab	5.4x10^6^ 1.4x10^5^, 2.5x10^7^	1.2x10^4^ 605, 2.4x10^6^	**0.019**	2.4X10^6^ 1.6x10^5^, 1.0X10^7^	1.7x10^4^ 605, 1.8x10^6^	**0.046**	0.13
***G*. *vaginalis***							
% (No.) PCR+	96% (49)	100% (51)	0.50	90% (18)	100% (19)	0.49	0.31
Gene copies/swab	2.49 x 10^6^ (1x10^5^, 1.3x10^7^)	2.33 x 10^6^ 1.96x10^5^, 8.7x10^6^	0.41	1.07 x 10^6^ 7.3x10^4^, 1.8x10^7^	1.04 x 10^6^ 9.4x10^3^, 5.0x10^6^	0.10	0.54
**BVAB2**							
% (No.) PCR+	72.5% (37)	68.6% (35)	0.82	85% (17)	84.2% (16)	>0.99	0.13
Gene copies/swab	181 25, 1.6x10^4^	55 25, 1.2x10^3^	0.69	2262 58, 1.0x10^5^	55 29, 7.2x10^3^	0.057	0.36
Prevotella bivia*							
% (No.) PCR+	31.1% (14/45)	28.3% (13/46)	0.82	35% (7/20)	16.7% (3/18)	0.28	0.83
Gene copies/swab	9.7 9.7, 99	9.7 9,7, 74	0.55	9.7 9.7, 2.2x10^3^	9.7 9.7, 9.7	0.21	0.70

Results reported as both number (No.) PCR+ and median gene copies/swab (IQR)

*Denominator for *L*. *iners* and *Prevotella bivia* differs from other species due to limitations in the quantity of DNA available.

## Discussion

The prevalence of HIV in postmenopausal women continues to increase as the life expectancy of HIV+ and HIV- women converge. Thus, it is important to characterize the cervicovaginal mucosal immune environment and vaginal microbiota in postmenopausal women as the two are likely linked and contribute to genital tract health as well as risk for HIV acquisition and transmission. The salient findings of this study are that HIV+ postmenopausal women have less CVL *E*. *coli* bactericidal activity, reflecting a lower proportion of lactobacilli species and a greater proportion of *Gardnerella* and *A*. *vaginae* and more HSV-2 inhibitory activity reflecting increased levels of inflammatory mediators compared to HIV+ premenopausal women. There is a wide distribution of vaginal Community State Types (CST) which has been described in detail for reproductive aged women [38] including those dominant in protective lactobacilli (*L*. *crispatus*, *L*. *gasserii*, or *L*. *jensenii*) (CST I, II or V) as well as those dominated by *L*. *iners* or diverse anaerobes (e.g. CST III or IV). CST IV species appear to be more prevalent in women who identify as Black or Hispanic suggesting race/ethnicity may be a factor mediating some of the variability in the vaginal microbiome of individual women [38–40]. Studies of postmenopausal women have also demonstrated marked variability in CST, with higher proportions of CST-IV species in women with vaginal dryness and/or atrophy.[1, 2] We did not collect data on symptoms of vaginal dryness or atrophy therefore we were unable to assess for this. Given the large proportion of study participants (55–67%) who reported their race as Black, it is reasonable to postulate that race may be one factor in a complex network of interactions mediating the vaginal microbiome in postmenopausal participants despite the lack of differences in alpha and beta diversity by self-reported race in this study (S4 Fig). The reduction in lactobacilli and concomitant increase in dysbiosis and inflammation that occur in the setting of menopause and HIV infection may merge to influence risk for genital tract HIV shedding and subsequent transmission (Fig 1).[41–44] The effect of menopause on mucosal immunity was greater in HIV+ compared to HIV- participants, suggesting a synergistic relationship likely reflecting the contribution of chronic immune activation that occurs with both aging and HIV infection.[21, 23, 45]

We focused on bactericidal activity of CVL against *E*. *coli* because it may be a functional biomarker of mucosal immunity and likely reflects cumulative interactions between molecules secreted by microbiota including lactic acid and bacterial surface proteins[13] and by human immune or epithelial cells. The relative contribution of these molecules may differ depending on the vaginal bacterial community state. In populations where lactic acid producing lactobacilli is dominant, *E*. *coli* bactericidal activity is relatively high, decreases in the setting of bacterial vaginosis (BV) and recovers with treatment.[46] In contrast, in populations where *L*. *iners* or diverse anaerobe community states (e.g. CST III or IV) are more prevalent including adolescence and, as shown here, HIV+ postmenopausal women, *E*. *coli* bactericidal activity tends to be relatively low and may be mediated more by human antimicrobial peptides such as defensins.[16, 47] In these settings, *E*. *coli* bactericidal activity may provide a surrogate biomarker for inflammation and may predict HIV risk as was suggested by a small study of HIV seroconverters in South Africa, where higher *E*. *coli* bactericidal activity was associated with increased HIV acquisition and positively correlated with HBD-1 and HBD-2.[9]

HSV inhibitory activity was highest in the HIV+ postmenopausal participants and correlated with host inflammatory proteins HNP1-3, lactoferrin, fibronectin, IL-6 and IL-8[16–18] consistent with prior studies. Inflammation has been linked to local genital tract HIV shedding[48, 49] however in the present study, differences were not detected in HIV shedding between pre and postmenopausal groups. This may reflect that only a few of the soluble proteins measured were significantly increased and CVL HIV shedding was only measured at a single time-point. Moreover, most of the women had suppressed plasma VL and were adherent to ART.

Multiple studies have shown that CVL may inhibit or enhance HIV infection *in vitro* although the precise mechanisms have not been elucidated and consistent associations have not been observed. [17, 50–52] In this study, CVL HIV inhibitory activity was higher in HIV+ compared to HIV- women, presumably reflecting HIV specific antibodies[53] and/or ART.[51, 54] The contribution of these factors might explain the lack of difference in HIV inhibitory activity when comparing postmenopausal vs. premenopausal HIV+ women since the majority were on suppressive ART. Paradoxically a substantial subset of participants displayed HIV enhancing activity of CVL despite ART. The inverse correlation between HIV inhibitory activity and quantities of *G*. *vaginalis* and BVAB2 is consistent with prior studies showing that HIV enhancing activity may be associated with BV.[55, 56] Genital tract drug levels were not measured in the current study, but enhancers were more likely to have detectable plasma VL, were more likely to be on PIs, which poorly penetrate the genital tract, and less likely to be on NNRTIs, which tend to have comparably higher genital tract penetration.[57] Previous studies have demonstrated increased rates of genital HIV shedding based on ART regimen, especially in participants on PI based regimens as compared to NNRTI based regimens (efavirenz) and in participants with increased immune activation and more advanced HIV disease stage.[22–24] It is feasible that inadequate penetration of ART into the genital tract or altered drug pharmacokinetics in the setting of dysbiosis and inflammation may permit low level viral replication, shedding of HIV in the vaginal compartment and potential for transmission.

HIV- postmenopausal women had significantly higher levels of HIV inhibitory activity compared to premenopausal women as previously reported in one study.[5] Other groups have demonstrated either no difference or less HIV inhibition in postmenopausal compared to premenopausal participants.[3, 58]. Discrepant results may reflect differences in HIV anti-viral assays used by others (Jurkat-Tat-CCR5)[58] as well as differences in populations studied. Further mechanistic studies are needed to identify the microbial or host factors that contribute to both genital tract HIV inhibitory and enhancing activity and to determine if this activity is of clinical significance.

There are several limitations to this study, including measurement of microbiota and immune mediators at a single time-point, lack of tissue to allow for assessment of gene expression, epithelial barrier integrity and immune cell populations, and absence of testing for other sexually transmitted infections including HPV or HSV. As the WIHS cohort is aging, we recruited a third of the premenopausal participants from outside the cohort. Because of this recruitment strategy, WIHS participants were older, more likely to be postmenopausal and have higher HSV inhibitory activity than the non-WIHS participants, however other variables were evenly matched between the groups. Despite these limitations, results suggest that menopause is associated with less *E*. *coli* bactericidal activity and more HSV inhibitory activity reflecting an altered, lactobacillus deficient vaginal microbiome and an increase in proinflammatory molecules and dysbiosis that might promote HIV shedding. Perhaps HIV+ postmenopausal women would benefit from an intervention such as topical estradiol or probiotics to improve the mucosal immune environment with the goal of improving genital tract health. The vaginal microbiome may modulate the inflammatory profile of the genital tract; this is supported by the finding that organisms commonly found in CST-IV (e.g. *Prevotella*, *Mobiluncus* and *Sneathia*) induce higher levels of proinflammatory cytokines including IL-1α, IL-1β and IL-8 compared to *L*. *crispatus* dominant CST-1 communities.[6, 20, 59] Further limited data suggest that symptoms of vaginal atrophy, a consequence of estrogen depletion in menopause may be partly mediated by vaginal dysbiosis and inflammation.[1, 2] Estrogen replacement has been shown to improve vaginal atrophy symptoms and the vaginal microbiome in HIV- postmenopausal women[60] suggesting that a similar intervention in HIV+ postmenopausal women may be beneficial.

In menopausal women living with HIV, shifts in the vaginal immune environment toward a CST IV microbiome and genital tract inflammation have increased importance given their association with genital tract HIV shedding and risk for transmission.[41–44, 48] Restoration of a more optimal, lactobacillus-dominant vaginal microbiome may have added benefits in menopausal women living with and at risk for HIV infection because the female genital tract microbiome has been shown to modulate tenofovir pharmacokinetics (PK).[61–63] Moreover, mucosal inflammation, which is linked to vaginal dysbiosis, has also been shown to alter ART efficacy within the female genital tract.[64] In a pilot study, treatment of HIV- postmenopausal women with vaginal estradiol cream was associated with a decrease in vaginal pH and proinflammatory cytokines and an increase in antimicrobial peptides.[3] Thus promotion of a lactobacillus dominant vaginal microbiome and reduced mucosal inflammation in HIV+ menopausal women may help alleviate symptoms of vaginal atrophy, improve genital tract health and reduce risk for shedding of HIV in the genital tract, which has the potential to fuel expansion of the viral reservoir and increase risk for HIV transmission.

## Supporting information

S1 Fig PD whole tree metric of alpha diversity by reproductive status and levels of *E*. *coli*, HIV and HSV inhibitory activity. Alpha diversity by reproductive status (A,B), varying levels of *E*. *coli* antimicrobial activity (C,D), HIV inhibitory activity (E,F), and HSV inhibitory activity (G,H) in vaginal secretions of HIV+ women (A,C,E,G) and HIV- women (B,D,F,H). All samples were rarefied at 7,690 sequences per sample. Significance was determined by an ANOVA test with resampling 999 times (p≤0.05).
(TIFF)

S2 Fig PD whole tree metric of alpha diversity (left panel) and ANOSIM test of beta diversity (right panel) of the vaginal microbiome among HIV+ women undergoing one of four antiretroviral therapies or no ART. (TIFF)

S3 Fig Beta diversity of vaginal communities by reproductive status and levels of *E*. *coli*, HIV and HSV inhibitory activity. Weighted UniFrac distances of vaginal communities between pre and postmenopausal participants (A,B), high, mid and low levels of *E*. *coli* antimicrobial activity (C,D), HIV inhibitory activity (E,F), and HSV inhibitory activity (G,H) in vaginal secretions of HIV+ women (A,C,E,G) and HIV- women (B,D,F,H). Significant differences in beta diversity were determined by an ANOSIM significance test with 999 sample permutations (p≤0.05).
(TIFF)

S4 Fig PD whole tree metric of alpha diversity and weighted Unifrac distance measure of beta diversity of the vaginal microbiome by Race. 100 HIV+ and 39 HIV- women self-identifying as Black, White or another race/ethnicity were included.
(TIFF)

S5 Fig Alpha diversity rarefaction curves by PD whole tree for HIV+ (A) and HIV- (B) participants. (TIFF)

S6 Fig QIIME Processing Workflow. (TIF)

S1 Table OTU Summary Table. Number of sequences and OTUs in HIV+ and HIV- Premenopausal and Postmenopausal Women.
(TIFF)
