# Supplementary figures and images for: Impact of reproductive aging on the vaginal microbiome and soluble immune mediators in women living with and at-risk for HIV infection
Source: PLoS One. 2019 Apr 26;14(4):e0216049. doi: 10.1371/journal.pone.0216049 (PMC6485713; doi:10.1371/journal.pone.0216049)

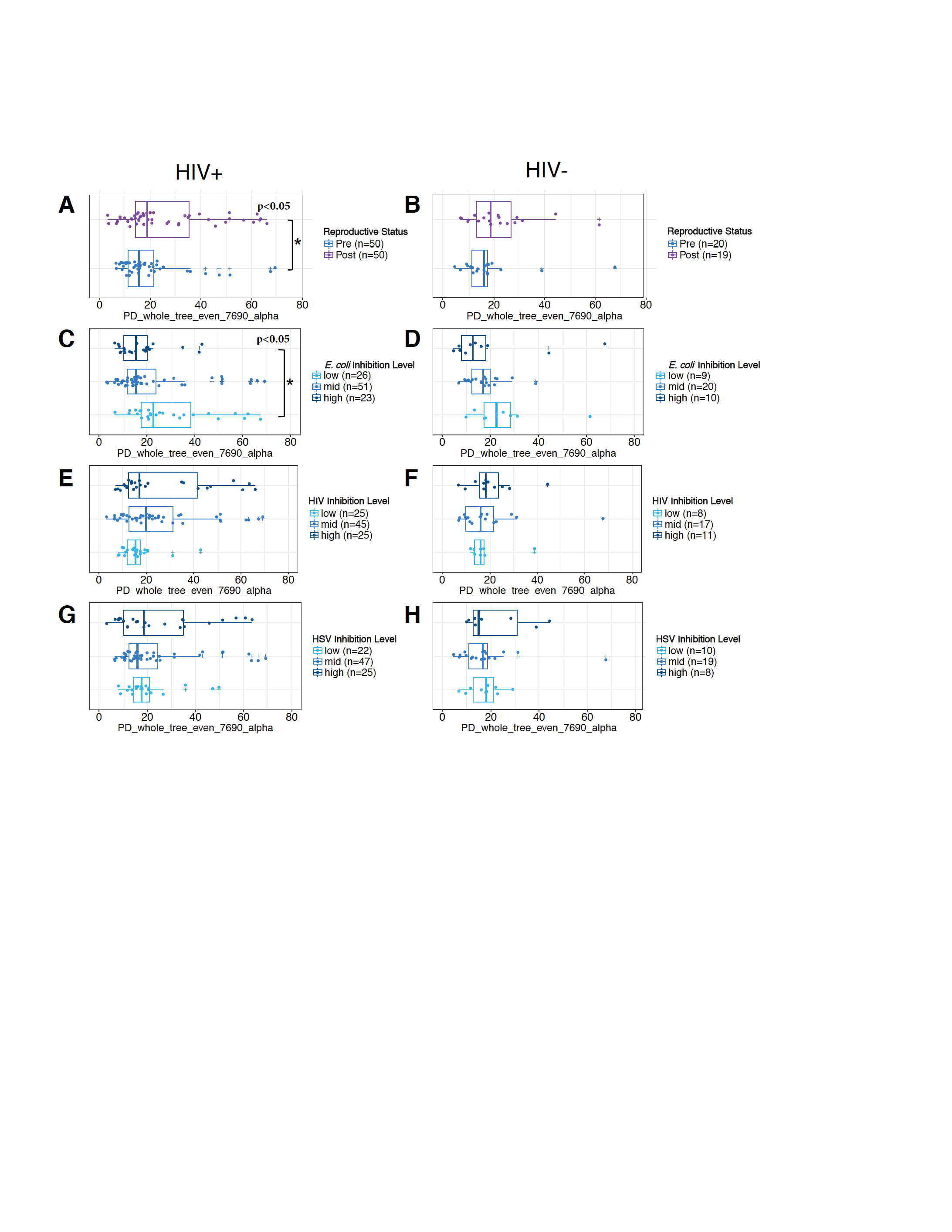

Supplement: S1 Fig — Alpha diversity by reproductive status (A,B), varying levels of E. coli antimicrobial activity (C,D), HIV inhibitory activity (E,F), and HSV inhibitory activity (G,H) in vaginal secretions of HIV+ women (A,C,E,G) and HIV- women (B,D,F,H). All samples were rarefied at 7,690 sequences per sample. Significance was determined by an ANOVA test with resampling 999 times (p≤0.05). (TIFF) [file pone.0216049.s001.tiff]

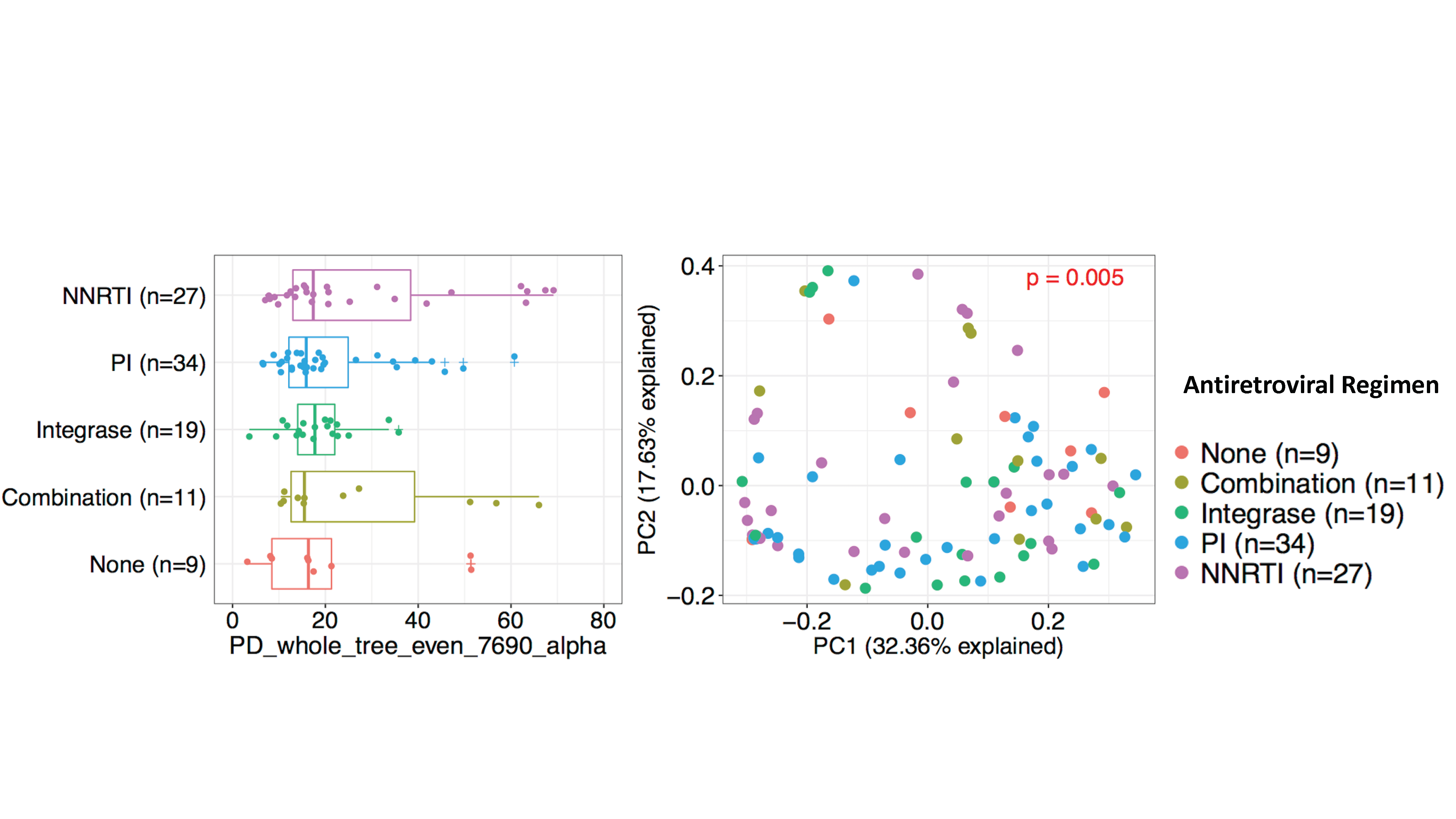

Supplement: S2 Fig — (TIFF) [file pone.0216049.s002.tiff]

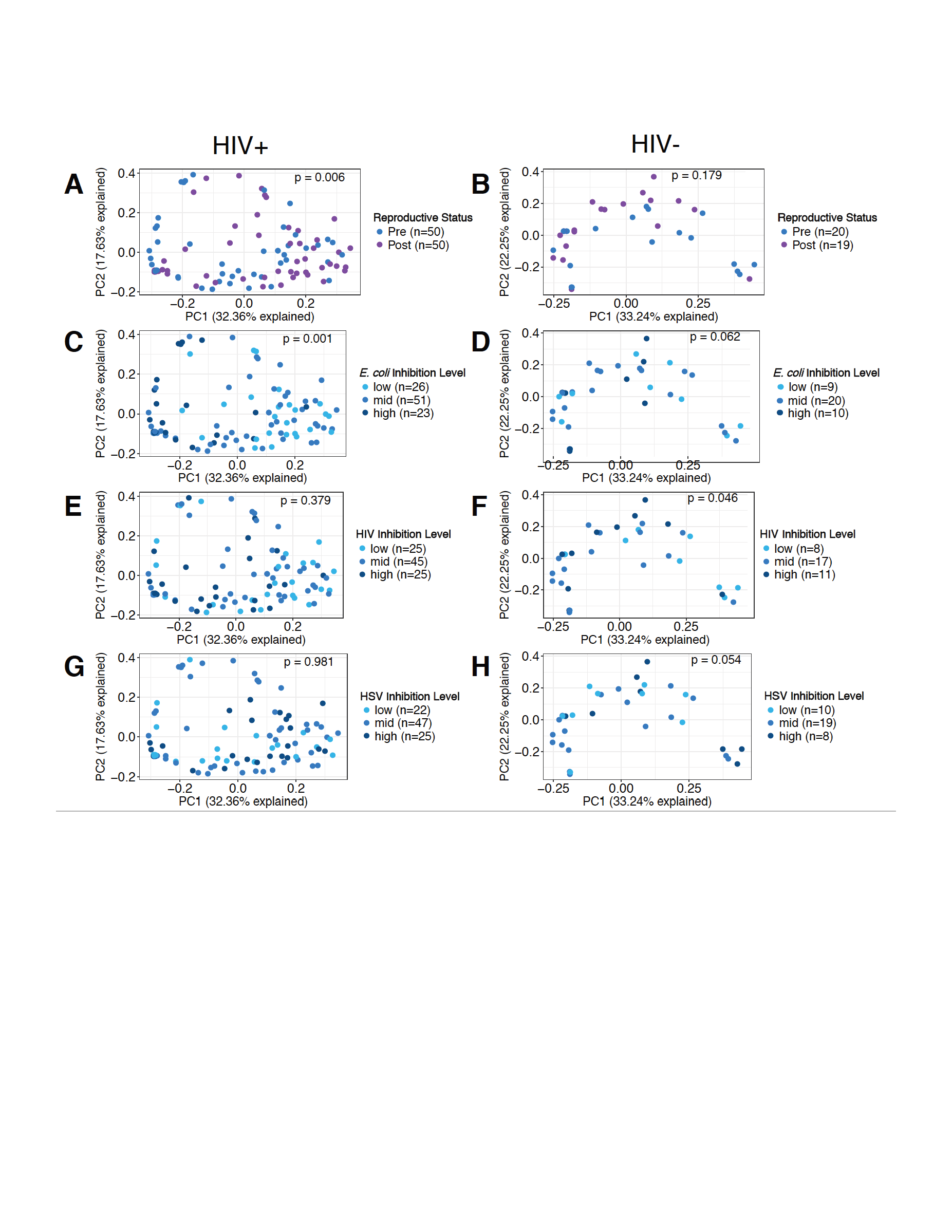

Supplement: S3 Fig — Weighted UniFrac distances of vaginal communities between pre and postmenopausal participants (A,B), high, mid and low levels of E. coli antimicrobial activity (C,D), HIV inhibitory activity (E,F), and HSV inhibitory activity (G,H) in vaginal secretions of HIV+ women (A,C,E,G) and HIV- women (B,D,F,H). Significant differences in beta diversity were determined by an ANOSIM significance test with 999 sample permutations (p≤0.05). (TIFF) [file pone.0216049.s003.tiff]

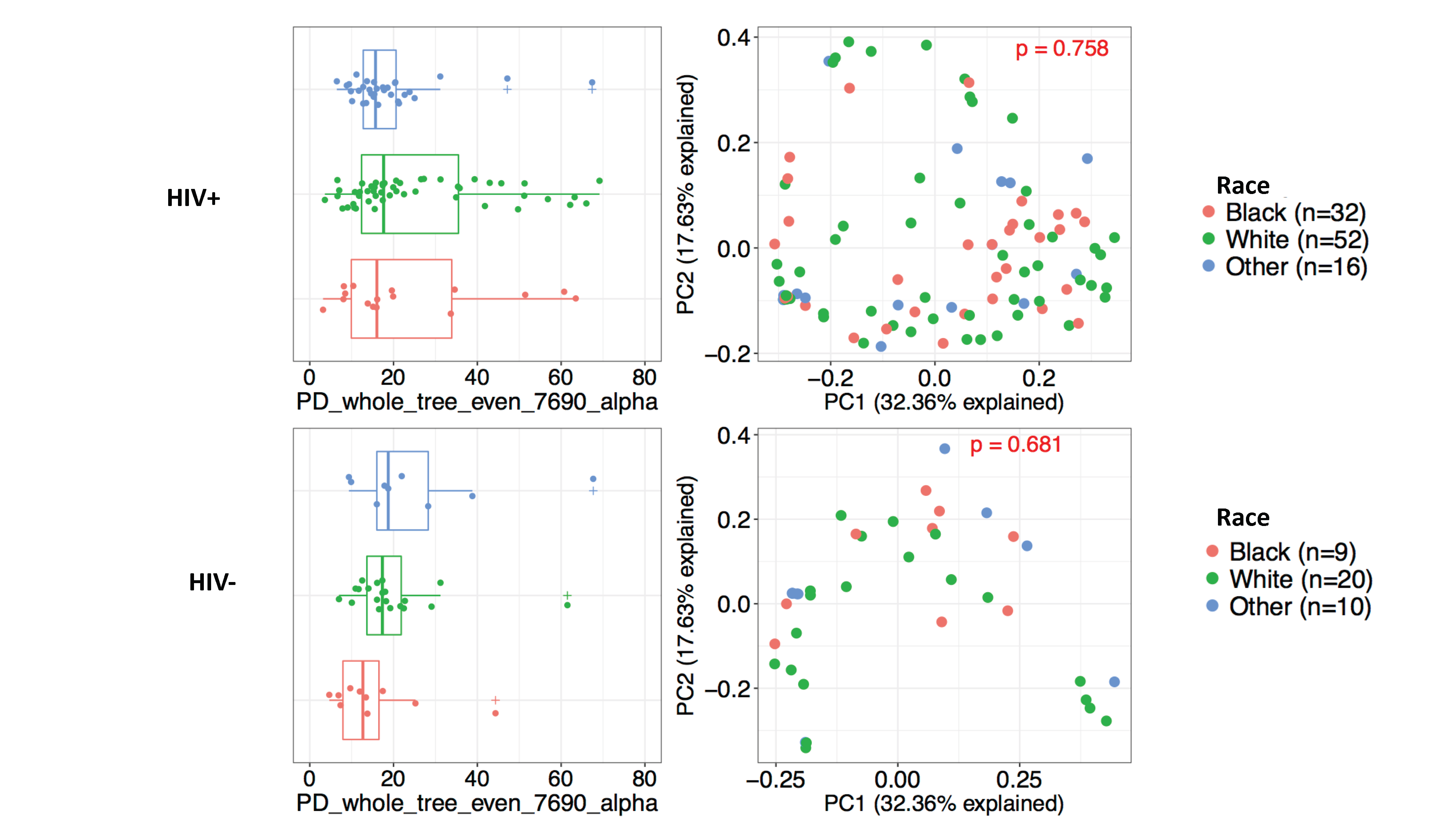

Supplement: S4 Fig — 100 HIV+ and 39 HIV- women self-identifying as Black, White or another race/ethnicity were included. (TIFF) [file pone.0216049.s004.tiff]

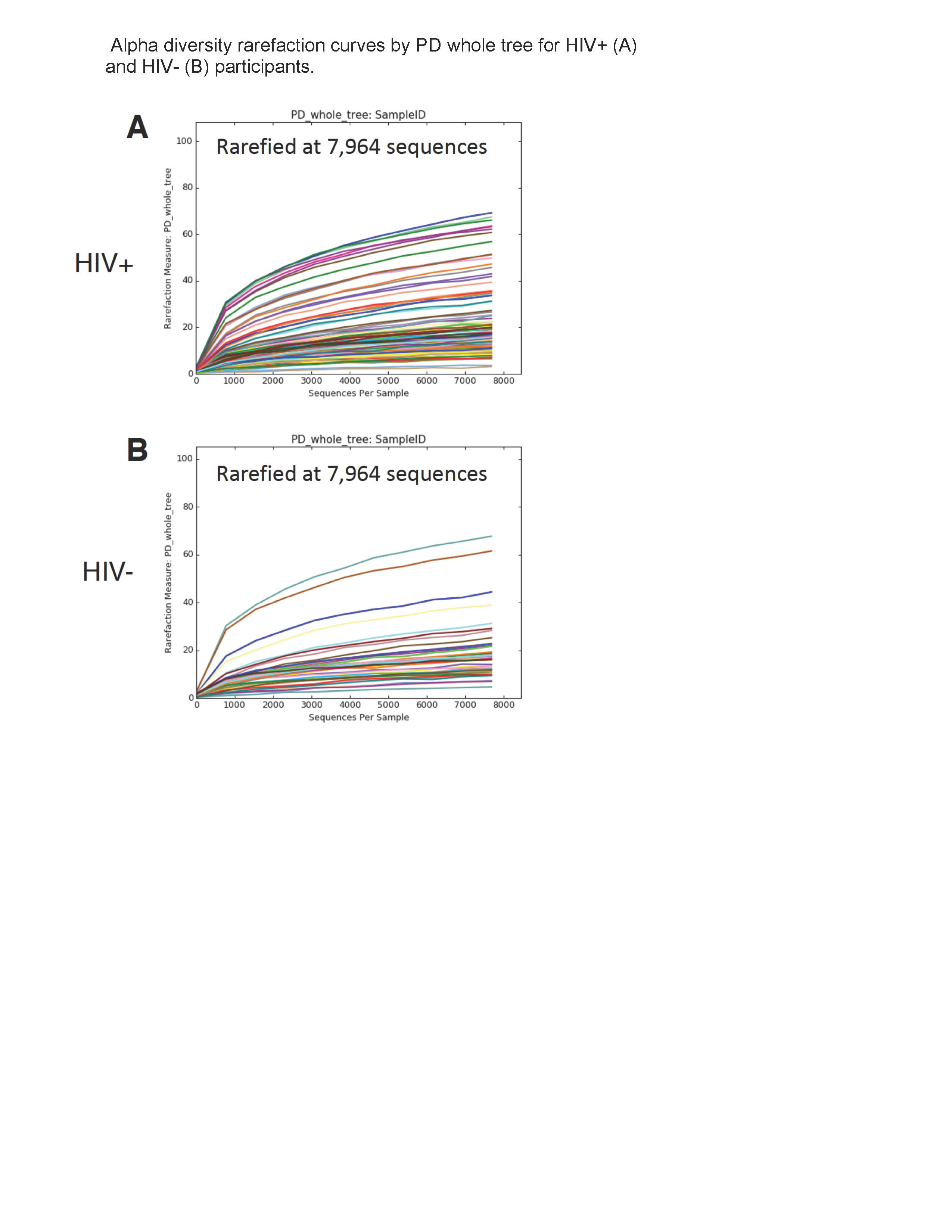

Supplement: S5 Fig — (TIFF) [file pone.0216049.s005.tiff]

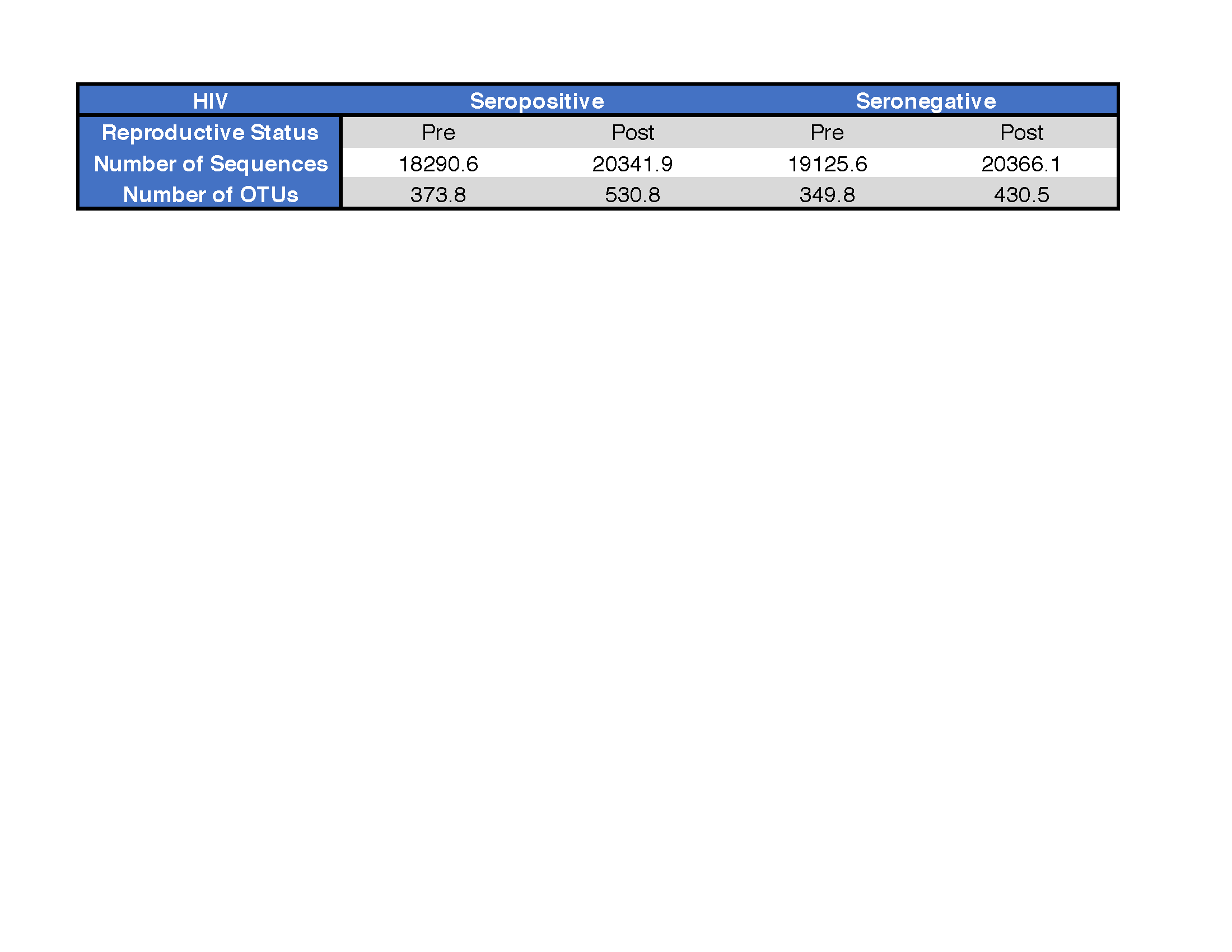

Supplement: S1 Table — Number of sequences and OTUs in HIV+ and HIV- Premenopausal and Postmenopausal Women. (TIFF) [file pone.0216049.s007.tiff]
